# Case of a Gun-Shot Wound Successfully Treated

**Published:** 1800-04

**Authors:** 


### *To the Editors of the Medical and Physical Journal.*†

GENTLEMEN,

THE following case having been noticed in the public papers, I have sent you a statement of the facts for your valuable Publication.

Lieut. Wynn, of the 17th foot, was landed at this place from the Helder, Nov. 3, with a wound in his side; and by the active benevolence of Major General Lennox,‡ placed immediately in a comfortable lodging, under the care of Mr. Melling, who has been surgeon to the garrison here upwards of forty years, with the able assistance of his very attentive partner Mr. Cranen, and under the direction of a physician, with whom the General would have intrusted himself had he been in similar circumstances.

---

† We received the following interesting case from a gentleman at Hull, which, we conceive, well merits the attention of all Surgeons in the Army and Navy. EDIT.

‡ The uncommon attention which this gentleman has always paid to the wants of the soldier under his command, has made him deservedly beloved by the army.

Mr. W. had been a fortnight at sea in bad weather, was extremely emaciated, very agitable and dyspeptic, having kept nothing upon his stomach whilst on board. He complained of slight cough, pain in the chest, and the region of the left kidney, but had no dyspnœa, and could lie on either side; his pulse was quick, and he was costive. He related that he had been wounded on the 2d of October, and he suspected by a grape-shot, for the party he commanded was within reach of a fort.

On examination after the accident, it appeared that the ball had entered the thorax by breaking the second false rib on the left side about the centre; but the surgeons at the Helder were not able to find the ball, nor to ascertain how far, or in what direction, it had penetrated; they however observed, that the flame of a candle was affected by the air as it passed in and out of the chest during respiration.

As soon as possible after he landed, every thing prudent was attempted in order to discover the ball; but it was not thought justifiable to comply with the earnest wishes of the patient, and to lay open the chest for the purpose of making a search into that cavity under the present circumstances.

The wound continued for some weeks to discharge a very large quantity of fœtid matter at every dressing; but during the night of the 24th, he was suddenly seized with a violent fit of coughing, and he expectorated a very large quantity of the same fœtid matter as the wound had hitherto discharged, which tasted, he thought, of iron; in the morning the wound was perfectly dry. A consultation was held, to determine what ought to be done; but as there was no clue to guide the operator, it was recommended to Mr. W. to wait with patience, till further information could be obtained, either from swelling, fixed pain, or other definite circumstance, which should point out the true situation of the ball.

The Medical Board having thought proper to send down Dr. Hunter to be surgeon to the sick and wounded Russians about that time, this gentleman requested permission to see the case; it was his opinion that the wound should be enlarged, and that further search should be made for the ball: but the objections to such an operation not being less than before, those who had hitherto constantly attended him, could only consider this as a matter of experiment. Accordingly, Dr. H. himself undertook the operation; having enlarged the wound between two and three inches, obliquely forwards and downwards, he examined it; but not being able to find the ball, nor any sinus that might lead to it, he concluded that the ball had not penetrated into the thorax, but had rebounded, and that the present inordinate spitting should be considered as phthisis. This opinion, however, ever, was never for one moment affented to by thofe who had hitherto devoted their time and attention to the cafe, full of confidence that at one time or other, an abfcefs might difcover the ball; Mr. Cranen continued his daily attentions, and his phyfician advifed the fame general plan of fupporting the patient.

On the night of Dec. 11, the cough ftopped as fuddenly as it had begun; and on taking off the dreffings, matter poured out of the wound, from a fmall opening near its lower edge; the probe was carefully introduced into this new aperture, and the ball was difcovered lying within the thorax, clofe to the broken rib, towards the fpine, between two and three inches from the fracture, and rather below the external opening.

Mr. W. was very impatient to have it extracted by the gentlemen prefent; but as Dr. H. was not there at the time when the ball was difcovered, the operation was fixed for the next day. Two large incifions were made by Dr. H. (who undertook to extract the ball); and having introduced the forceps between the ends of the broken rib, he fucceeded in laying hold of it, but could not get it further than the opening formed by the fracture, the ball receding from the forceps into the cavity every time force was employed to draw it through. On the arrival of further affiftance, a portion of the broken rib was immediately advifed to be removed, in order to give room for the ball to pafs; this was accordingly done. Upon again bringing it forward, the opening was ftill found lefs than the ball, and that the forceps, (intended only for a leaden bullet) could not fufficiently grafp and retain their hold of the ball, to extact it; a lever was therefore introduced behind the ball, and it was forced out by that means.

Notwithftanding Mr. W. was upwards of half an hour under the operation, which he bore with uncommon fortitude, he had no untoward fymptoms, except diarrhœa; no cough of any confequence, no inflammation of the pleura; the wound healed to all appearance as well as if placed in any other part of the body; and when he left this place, which he did on March 1, in apparent health and high fpirits, there remained only a very trifling opening, probably occafioned by a fmall exfoliation of the rib. After the operation, the flame of a candle applied to the wound was affected by refpiration.

The ball is of caft-iron, and weighs three ounces and a half.

I am,

Gentlemen,

Your's, &c.

