# Errata

**Published:** 1800-05

**Authors:** 


					ERRATA.
Vol. HI. p. 35Jand S55, for Mr. Cm?^. read itfh. Craven.

				

