# Errata

**Published:** 1800-05

**Authors:** 


					ERRATA.
. Vol. m. p. 35J and 555, for Mr. CraxertK vend Afh Craven*

				

